# The CBL-Interacting Protein Kinase NtCIPK23 Positively Regulates Seed Germination and Early Seedling Development in Tobacco (*Nicotiana tabacum* L.)

**DOI:** 10.3390/plants10020323

**Published:** 2021-02-08

**Authors:** Sujuan Shi, Lulu An, Jingjing Mao, Oluwaseun Olayemi Aluko, Zia Ullah, Fangzheng Xu, Guanshan Liu, Haobao Liu, Qian Wang

**Affiliations:** 1 Tobacco Research Institute, Chinese Academy of Agricultural Sciences, Qingdao 266101, China; shisujuan2014@163.com (S.S.); lulu_an9@163.com (L.A.); maojingjing40@163.com (J.M.); aluko.oluseun@gmail.com (O.O.A.); zianust512@gmail.com (Z.U.); xufangzheng@caas.cn (F.X.); liuguanshan@caas.cn (G.L.); 2 Graduate School of Chinese Academy of Agricultural Sciences (CAAS), Beijing 100081, China; 3 Technology Center, Shanghai Tobacco Co., Ltd., Beijing 101121, China

**Keywords:** *CIPK23*, seed germination, cotyledon expansion, hypocotyl elongation, seedling growth, tobacco

## Abstract

CBL-interacting protein kinase (CIPK) family is a unique group of serine/threonine protein kinase family identified in plants. Among this family, *AtCIPK23* and its homologs in some plants are taken as a notable group for their importance in ions transport and stress responses. However, there are limited reports on their roles in seedling growth and development, especially in *Solanaceae* plants. In this study, *NtCIPK23*, a homolog of *AtCIPK23* was cloned from *Nicotiana tabacum*. Expression analysis showed that *NtCIPK23* is mainly expressed in the radicle, hypocotyl, and cotyledons of young tobacco seedlings. The transcriptional level of *NtCIPK23* changes rapidly and spatiotemporally during seed germination and early seedling growth. To study the biological function of *NtCIPK23* at these stages, the overexpressing and CRISPR/Cas9-mediated knock-out (*ntcipk23*) tobacco lines were generated. Phenotype analysis indicated that knock-out of *NtCIPK23* significantly delays seed germination and the appearance of green cotyledon of young tobacco seedling. Overexpression of *NtCIPK23* promotes cotyledon expansion and hypocotyl elongation of young tobacco seedlings. The expression of *NtCIPK23* in hypocotyl is strongly upregulated by darkness and inhibited under light, suggesting that a regulatory mechanism of light might underlie. Consistently, a more obvious difference in hypocotyl length among different tobacco materials was observed in the dark, compared to that under the light, indicating that the upregulation of *NtCIPK23* contributes greatly to the hypocotyl elongation. Taken together, *NtCIPK23* not only enhances tobacco seed germination, but also accelerate early seedling growth by promoting cotyledon greening rate, cotyledon expansion and hypocotyl elongation of young tobacco seedlings.

## 1. Introduction

Calcium (Ca^2+^) is a ubiquitous second messenger in the plant. When plants are stimulated by environmental and developmental changes, the concentrations of intracellular Ca^2+^ changes spatially and temporally, and form diverse calcium signals that are sensed and decoded by different calcium sensors [1]. Among the sensors, the Calcineurin B-like protein (CBL) family plays an important role in plant responses to stimuli [2,3]. CBLs always interact with CBL-interacting protein kinase (CIPK) family to form a complicated but flexible CBL-CIPK network [3,4]. The latter participates in the regulation of plant responses to biotic and abiotic stresses, through the phosphorylation of downstream target proteins, thus subsequently influencing their activities [5]. CIPK family is a plant-specific class of serine/threonine protein kinase family, which was also classified as Group 3 of the sucrose non-fermenting 1-related kinases (SnRK3) [6]. The CIPK family is the key factor linking the upstream Ca^2+^ signals to downstream targets in plant stress response signaling pathways [2]. Generally, CIPKs are structurally conserved, possessing an *N*-terminal kinase catalytic domain, and a *C*-terminal regulatory domain harboring a NAF/FISL motif and a phosphatase interaction motif. CIPKs interact with the CBLs via their NAF/FISL module [7].

Many CIPK family members from different plant species, including *Arabidopsis* [5], rice [8], maize [9], wheat [10], and soybean [11] were isolated and some are deeply elucidated. Among these members, AtCIPK23 and its homologs (here we refer to them simply as CIPK23s) are more notable, due to their roles in the regulation of plant responses to abiotic and biotic stresses. Generally, the functions of CIPK23s in these processes are established by its regulation in ion transport. In *A*. *thaliana*, two pathways involved in potassium signaling cascade; AtCBL1/9-AtCIPK23-*Arabidopsis* K^+^ Transporter 1 (AKT1) and AtCBL1-AtCIPK23-High-Affinity K^+^ Transporter 5 (AtHAK5) pathway, were identified to positively regulate K^+^ acquisition under low K^+^ condition [12,13,14,15]. Similarly, the OsCBL1-OsCIPK23-OsAKT1 and VvCBL1-VvCIPK4-K^+^ Channel (VvK1.2) pathways were also characterized in rice (*Oryza sativa*) [16] and grape (*Vitis vinifera*) [17], respectively. Under high external nitrate (NO_3_^−^) concentration, the AtCBL1/9-AtCIPK23-Nitrate Transporter 1.1 (AtNRT1.1/CHL1) pathway and the AtCBL9-AtCIPK23-Nitrate Transporter 2.1 (AtNRT2.1) pathway were reported to inhibit NO_3_^-^ transport [18,19]. Under low external nitrate conditions, the AtCBL1/9-AtCIPK23-AtCHL1 pathway positively regulates NO_3_^-^ transport [18]. When the *Arabidopsis* roots were exposed to high ammonium (NH_4_^+^) conditions, AtCIPK23 leads to the allosteric inactivation of high affinity Ammonium Transporter 1 (AMT1) through phosphorylation, and subsequently inhibits NH_4_^+^ transport, thus protecting the plants from NH_4_^+^ toxicity [20]. In our recent work, *AtCIPK23* is strongly upregulated in leaves and roots, significantly alleviates NH_4_^+^ toxicity triggered by high NH_4_^+^/K^+^ ratio, and reduces the leaf chlorosis and root growth inhibition by regulating the contents of NH_4_^+^ and K^+^ in these tissues [21]. Under excessive magnesium (Mg^2+^) stress, AtCBL2/3 interact with AtCIPK3/9/23/26, to sequester Mg^2+^ into the vacuole and protect plants from Mg^2+^ toxicity [22]. AtCIPK23 also regulates the stomatal closure by controlling anion and K^+^ efflux under drought stress by forming AtCBL1/9-AtCIPK23 complex to activate Slow Anion Channel Associated 1 (SLAC1) and Slow Anion Channel 1 Homolog 3 (SLAH3) [23,24]. Recently, the CIPK23 protein was also identified to participate in biotic stress responses. In cassava (*Manihot esculenta*), MeCBL1/9-MeCIPK23 positively regulates plant defense response to *Xanthomonas axonopodis* pv. *Manihotis* [25]. *OsCIPK23* was found to be mainly expressed in pistil and anther, and is up-regulated during pollination. Additionally, the pollen grains of *OsCIPK23*-RNAi lines were irregularly shaped or pear-shaped and contained a large empty central vacuole without any starch granules, resulting in sterility and reduced seed set [26]. Through a sensitivity analysis of *atcipk23* seeds to ABA, *AtCIPK23* was found to function in seed dormancy and germination of *A*. *thaliana* [27], indicating that ABA signaling might be enhanced in *AtCIPK23* loss-of-function materials. A recent study indicated that, AtCIPK23 regulates blue light-dependent stomatal opening in *A*. *thaliana* through activation of K^+^_in_ channels [28].

Although the functions of CIPK23s were extensively investigated in *A*. *thaliana* and some other plants. However, there are very few reports about their roles in plant growth and development, especially in *Solanaceae* plants, most of which are economically important. Tobacco is an ideal model plant in the gene functional research of solanaceous plants. In this study, *NtCIPK23*, a homolog of *AtCIPK23*, was cloned from *Nicotiana tabacum* L. cv. Zhongyan 100 (ZY100), and its tissue expression analysis during the seedling emergence was initially analyzed in detail. To identify its biological function, tobacco materials with different expression levels of *NtCIPK23* were obtained and comparative phenotypic analysis during the early seedling growth and development was then performed. The results might provide new clues to unveil the biological functions of CIPK23s in solanaceous plants and be of considerable importance for crop production.

## 2. Results

### 2.1. Sequence Analysis and the Subcellular Localization of NtCIPK23

**Figure 1:**
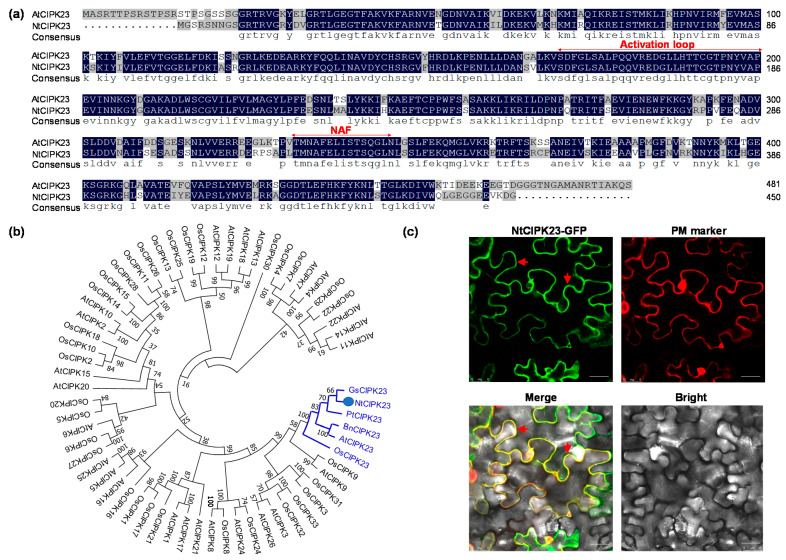
Sequence analysis and subcellular localization of NtCIPK23. (**a**) Amino acid alignment of NtCIPK23 with AtCIPK23. Identical and similar amino acids are shaded black and grey, respectively. The kinase activation loop and the NAF motif, which is named by the conserved amino acids Asn (N), Ala (A), and Phe (F) and is critical for the CBL-CIPK interaction, are also displayed. (**b**) Phylogenetic analysis of NtCIPK23 and CIPKs in *Arabidopsis*, rice, and other plants. At and Os represent *A. thaliana* and *O. sativa*, respectively. (**c**) Subcellular localization of NtCIPK23 in the epidermal cells of *N. benthamiana* leaves. The red arrows refer to PM. PM marker (pm-rk *CD3*-*1007* plasmid) is *A. thaliana* fatty acid desaturase 8 (AtFAD8) fused with red fluorescent protein mCherry. AtFAD8 is located in plasma membrane and chloroplast envelope. Scale bar is 25 μm.

Based on the bioinformatic analysis, the homolog of *AtCIPK23* (GenBank No. XM_016594430.1) was cloned directly from *N. tabacum* L. cv. ZY100 and was designated as *NtCIPK23*. NtCIPK23 shares 83.56% amino acid sequence similarity with AtCIPK23. Similar to other CIPK proteins, the NtCIPK23 protein harbors the conserved activation loop and NAF motif that is necessary to bind CBL proteins (Figure 1a) [5]. Phylogenetic analysis indicated that *CIPK23* gene is conserved during species evolution, and NtCIPK23 is on the same branch with AtCIPK23 and other CIPK23s, in the phylogenetic tree (Figure 1b).

In plants, subcellular localization analysis of a protein can provide useful clues for its functional identification. It was found that, AtCIPK23 and OsCIPK23 are located at the plasma membrane (PM) and play a key role in ion transport, mainly by phosphorylating some PM-located channels and transporters [15,16]. To identify the subcellular localization of NtCIPK23, a plasmid expressing NtCIPK23 fused with green fluorescent protein (GFP) at its C terminus (NtCIPK23-GFP) was constructed and introduced into the epidermal cells of *N*. *benthamiana* leaves. Confocal fluorescence microscopy analysis indicated that the strong GFP signal of NtCIPK23-GFP was detected mainly at the PM of the epidermal cells, which coincided with the PM marker pm-rk *CD3*-*1007* plasmid fused with red fluorescent protein mCherry [29] (Figure 1c). While a fraction of GFP signal was also detected in the cytoplasm and nucleus. As a negative control, a diffuse pattern of fluorescence that was both nuclear and cytoplasmic was observed in the cells expressing free GFP (data not shown). The results indicated that NtCIPK23 is mainly located on the PM (Figure 1c). It might act as other CIPK23s and mainly function at the PM to phosphorylate some PM-located targets [30].

### 2.2. Expression Pattern of NtCIPK23 during Seed Germination and Early Seedling Growth

**Figure 2:**
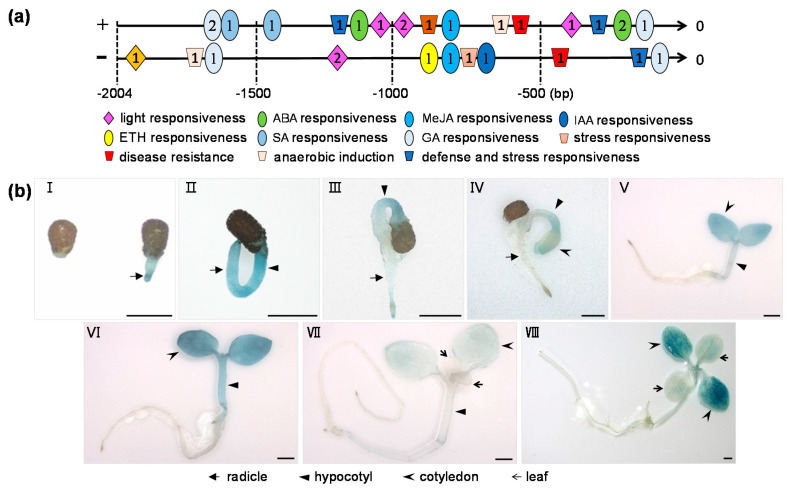
Expression pattern analysis of *NtCIPK23*. (**a**) The schematic distribution of *cis*-acting elements of *NtCIPK23* promoter. The *cis*-acting elements were predicted by the online software PlantCARE (http://bioinformatics.psb.ugent.be/webtools/plantcare/html/). Different colors and shapes represent different *cis*-acting elements. The characters in the graph indicate the number of predicted elements. “+” and “-” represent the sense and antisense strand, respectively. (**b**) The GUS staining result at different growth stages of *ProNtCIPK23*::*GUS* transgenic plants. The stages include micropylar endosperm rupture and radicle emergence at 3 DAS (I), radicle elongation (II) and hypocotyl elongation during 3~3.5 DAS (III), cotyledon emergence at 3.5~5 DAS (IV), cotyledon expansion during 5~6 DAS (V), cotyledon maturation during 6~8 DAS (VI), emergence of the first two leaves at 10 DAS (VII), and expansion of the first two leaves at 14 DAS (VIII). The experiment was performed using three independent repeats (*n* ≥ 9 plants). Scale bar is 0.5 cm.

As bioinformatic analysis of the native promoter always provides new starting points for the functional characterization of a gene, here, a 2004 bp promoter segment upstream of the start codon of NtCIPK23 was obtained from ZY100, based on the information provided by the NCBI Database (https://www.ncbi.nlm.nih.gov/nuccore). The *cis*-acting elements of NtCIPK23 promoter were then predicted by the online software PlantCARE (http://bioinformatics.psb.ugent.be/webtools/plantcare/html/). Besides the eukaryotic transcriptional regulatory elements (TATA-box and CAAT-box), there are other kinds of *cis*-acting elements distributed in the promoter, including light response elements, hormone response elements, anaerobic response elements, and stress defense-related components (Table S1). The number and relative positions of these *cis*-acting elements are shown in Figure 2a. The analysis indicated that the transcription of *NtCIPK23* might be regulated by various environmental signals, such as light, hormone, and some stresses, which hinted that *NtCIPK23* might contribute to the growth and developmental processes in tobacco plants.

A GUS staining assay was then conducted to study the tissue expression of *NtCIPK23* during seedling germination and early developmental stages, using the *ProNtCIPK23*::*GUS* transgenic lines. Evident GUS activity was detected in the radicle and hypocotyl when the testa was ruptured and the radicle was exposed (Figure 2b(I,II)). During the process of hypocotyl elongation and cotyledon emergence, a slight decrease of GUS activity was observed in the hypocotyl and nascent cotyledons, while no obvious activity was detected in the radicle tissue (Figure 2b(III,IV)). At the expansion stage of cotyledons, strong GUS activity was detected in the hypocotyl and two cotyledons (Figure 2b(V)), and when the cotyledons are fully expanded, GUS activity in the hypocotyl and cotyledons was at its peak (Figure 2b(VI)). After emergence of two leaves, the GUS activity in the hypocotyl and cotyledons declined rapidly to a much lower level, and no obvious activity was detected at the two young leaves (Figure 2b(VII)). Interestingly, it was observed that, during the growth of the two leaves, strong GUS activity in two cotyledons was recovered to a higher level (Figure 2b(VIII). GUS staining assay indicated that a series of spatiotemporal changes of *NtCIPK23* occur between the seed germination and early seedling developmental stages, suggesting that *NtCIPK23* transcription might be controlled under a sophisticated regulatory network.

### 2.3. NtCIPK23 Plays a Positive Role in Seed Germination and Post-Germination Seedling Growth under Normal Conditions

**Figure 3:**
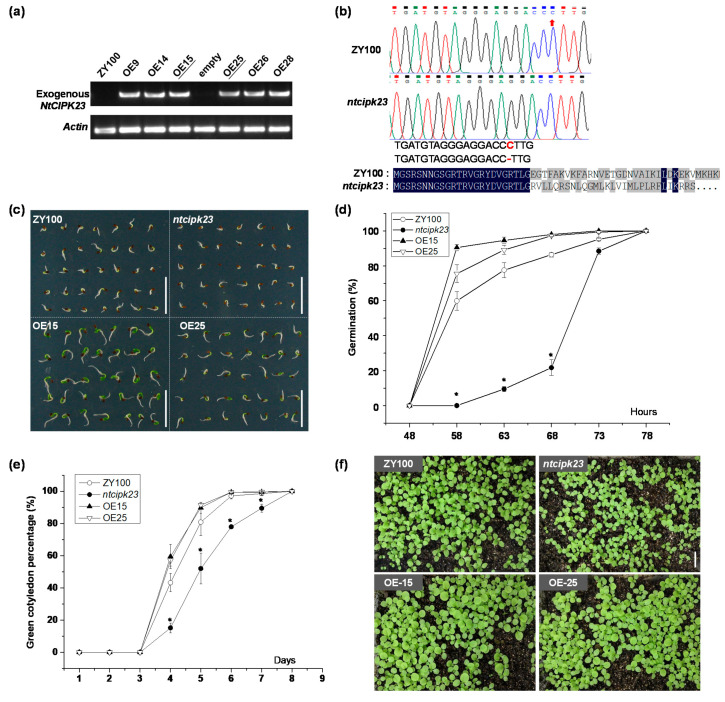
Seed germination and post-germination seedling growth of tobacco materials with different *NtCIPK23* expression levels. (**a**) The expression analysis of exogenous *NtCIPK23* in different tobacco materials. Here, primers specifically amplifying the exogenous *NtCIPK23* were designed elaborately, according to the sequences of *NtCIPK23* CDS and the transformation vector. As expected, bright bands were obtained in *NtCIPK23* overexpressing lines, while no signals were detected in wild type ZY100. (**b**) C deletion at the editing site in *NtCIPK23* gene of *ntcipk23* plants. “-” indicates the “C” deletion at position 67, which leads to an early translation termination. (**c**) Germination phenotype of the tobacco materials. (**d**) Germination percentage of the tobacco materials. (**e**) Green cotyledon percentage of the tobacco materials. (**f**) The seedling emergence and growth of tobacco materials with different *NtCIPK23* expression levels (20 DAS). All data are shown as the mean ± SE. *n* ≥ 100, independent samples collected from four experiments, and they were analyzed using the one-way ANOVA, where the threshold of significance is indicated above (* *p* < 0.05). Scale bar is 1.0 cm.

Evident GUS activity in the radicle and hypocotyl during germination and early seedling growth stages implied that *NtCIPK23* might function in this process. To clarify its role, the overexpressing and loss-of-function mutant lines of *NtCIPK23* were generated, respectively. Two overexpressing lines (OE15 and OE25, Figure 3a) and one typical mutant line, *ntcipk23*, were selected for the subsequent phenotype analysis. The *ntcipk23* mutant line was obtained by the CRISPR-Cas9 technique (Figure S1), and the C deletion at position 67 of *NtCIPK23* CDS results in a frameshift at the 5′-terminal region of its transcripts and leads to a subsequent translation termination (Figure 3b, Figure S2).

Germination rate and green cotyledon percentage of these materials under normal growth conditions were evaluated. Generally, the radicles of ZY100 seedlings normally break through seed coat within 3 DAS, and the cotyledons then emerge and turn green 2~4 days later. The seeds of overexpressing lines germinated more rapidly and the radicles elongated at a higher rate, compared to the wild type ZY100, while *ntcipk23* seeds germinated more slowly and the radicles elongated at a lower rate, although they all germinated eventually (Figure 3c,d). Green cotyledon percentage of these materials was then evaluated for post-germination seedling growth. No obvious difference was observed in the time taken for the cotyledon to emerge and the percentage of both ZY100 and overexpressing lines (Figure 3e), which might be triggered by the relative higher expression level in the hypocotyl in wild type plants. At 8 DAS, all seeds of the four plant materials germinated well. The result demonstrated that *NtCIPK23* plays a positive role in the process of seed germination and post-germination seedling growth, under normal growth conditions, and knock-out of the gene might affect seed vigor but not the ability to germinate (Figure 3f).

### 2.4. Overexpression of NtCIPK23 Promotes the Cotyledon Expansion of Young Tobacco Seedlings

**Figure 4:**
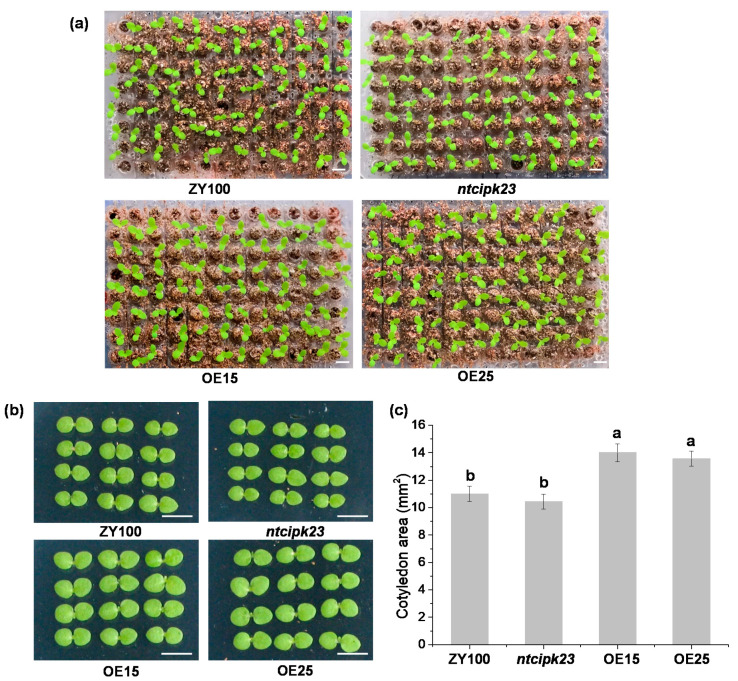
The phenotyping and data analysis of the cotyledon area of different tobacco materials. (**a**) Tobacco plants with different cotyledon size at 8 DAS. Scale bar is 0.5 cm. (**b**) Cotyledons of different tobacco materials. Scale bar is 0.5 cm. (**c**) The analysis of cotyledon area of different tobacco materials. Different lowercase letters (a and b) indicate significant differences at *p* < 0.05 according to the LSD test. The data are shown as the mean ± SE. *n* = 24, independent samples collected from three experiments.

Strong GUS activity was observed in the nascent cotyledons, so the cotyledon growth of different tobacco materials was observed. It was found that, compared to ZY100, the overexpressing lines possessed larger cotyledons, while those of *ntcipk23* were smaller (Figure 4a). When the cotyledons were fully expanded and the leaves emerged, the cotyledon area of each material was measured. The cotyledon area of *NtCIPK23*-overexpressing lines was significantly larger than that of ZY100, while the area of *ntcipk23* was indicated to be slightly smaller (Figure 4b,c). The data indicated that overexpression of *NtCIPK23* promotes the cotyledon expansion of tobacco seedlings.

### 2.5. NtCIPK23 Positively Regulates the Hypocotyl Elongation of Young Tobacco Seedlings

**Figure 5:**
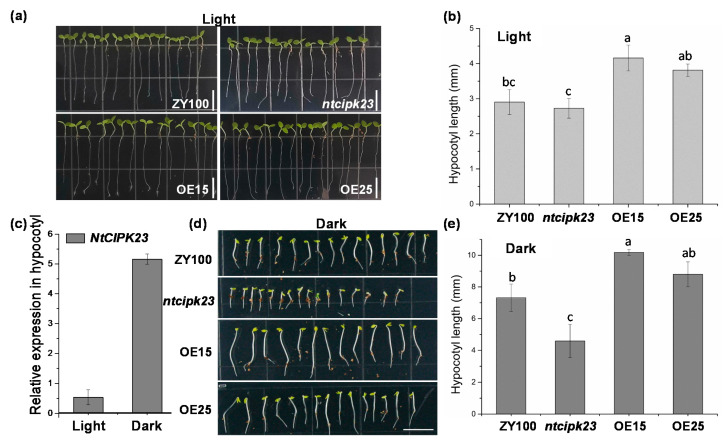
The phenotype and data analysis of hypocotyl in tobacco materials with different *NtCIPK23* expression levels. (**a,b**) Hypocotyl phenotype of different tobacco materials under light. (**c**) Expression of *NtCIPK23* in the hypocotyl of wild type ZY100 seedlings under the light and dark conditions. The relative transcript levels were normalized to the abundance of reference gene *NtL25*. (**d,e**) Hypocotyl phenotype of different tobacco materials in the dark. The plants under dark (wrapped by aluminum foil) were taken out at 6 DAS. Different lowercase letters (**a**–**c**) indicate significant differences at *p* < 0.05 according to the LSD test. The data are shown as the mean ± SE. *n* ≥ 20 plants, independent samples collected from three experiments. Scale bar is 1.0 cm.

Strong GUS activity was observed in the tobacco hypocotyl during seed germination, so the hypocotyl length of different tobacco materials was quantified. It was found that, under constant light, the hypocotyl length of these two overexpressing lines was the longest, followed by the wild type ZY100, and the *nicipk23* mutant possessed the shortest hypocotyl, indicating the promotive function of *NtCIPK23* in hypocotyl elongation (Figure 5a,b). As the crucial function of light in hypocotyl elongation and the distribution of some light-responsive *cis*-acting elements was predicted in the *NtCIPK23* promoter, we investigated the influence of light on *NtCIPK23′*s expression by GUS staining (Figure S3) and qRT-PCR (Figure 5c). It was shown that the expression of *NtCIPK23* in hypocotyl in the dark treatment was at a higher level, which was about ten times more than that under the light. The gene might be involved in the light signaling pathway by being inhibited by light and upregulated in the dark. To further analyze the role of *NtCIPK23* in hypocotyls, a germination experiment under dark conditions was performed. It was found that a more evident difference of hypocotyl length between *ntcipk23* and ZY100 was observed than that under the light, which means the upregulation of *NtCIPK23* triggered in the dark promotes the hypocotyl elongation (Figure 5d,e). Consistently, the hypocotyl length of *NtCIPK23*-overexpressing lines was also significantly longer than that of ZY100 (Figure 5d,e). Taken together, *NtCIPK23* works as a positive regulator in the process of hypocotyl elongation.

## 3. Discussion

To date, CIPK23 was found to act as a major regulator driving root responses to diverse environmental stimuli, including drought, salinity, and nutrient imbalances [31,32,33]. However, only a few investigations were conducted to characterize their roles in plant normal growth and development. Moreover, there are few reports about *CIPK23* genes in *Solanaceae.* In this study, a solanaceous *CIPK23*, *NtCIPK23*, was cloned from *N. tabacum* and its function in tobacco growth and development was first characterized. Through the analysis of expression pattern and phenotyping of tobacco lines with different *NtCIPK23* expression levels, *NtCIPK23* was found to enhance seed germination and early seedling development in tobacco.

For most dicotyledonous plants, cotyledon is the main storage organ that provides nutrients for seed germination and early seedling growth, and it is also the first organ for photosynthesis after germination [34]. Therefore, cotyledon plays a critical role in the early stage of seed germination and seedling growth. Here, it was found that the expression level of *NtCIPK23* was dramatically enhanced during cotyledon greening and reached a peak when the cotyledons were fully expanded (Figure 2b(V,VI)). Consistently, seed germination rate and cotyledon greening rate, as well as the cotyledon size, were all shown to be related to the relative expression level of *NtCIPK23* (Figure 3 and Figure 4). The results hinted that *NtCIPK23* might function as an activator to facilitate nutrient conversion, chloroplast development or photosynthesis establishment, and thus positively promote seed germination, cotyledon extension, and greening.

*NtCIPK23* was abundantly expressed in hypocotyl, and its expression level was greatly upregulated in dark treatment (Figure 2 and Figure 5c, Figure S3). Obvious inhibition of hypocotyl elongation in the *ntcipk2*3 mutant was observed (Figure 5a). Hypocotyl is the structure connecting root, shoot tip, and leaves in young seedlings. Its elongation is a critical growth stage for the epigaeous seedlings, to geminate in the dark in soil and reach for light [34]. Emergence capacity and emergence time of a seedling are strongly influenced by its hypocotyl length and the elongation speed [35]. Based on the knowledge of *AtCIPK23* in ion uptake or transport [14,15,20,21], *NtCIPK23* might promote hypocotyl elongation and seedling emergence by interfering in cell turgor and cell elongation by regulating ion absorption or transport.

Thus far, a wide variety of nutrient transporters were characterized to be the regulatory targets of AtCIPK23, including AKT1, AtHAK5, AtKUP4, AtNRT1.1, AMT1.1, SLAC1, SLAH3, etc. [31,36]. Through interfering their activity, the kinase regulates plant response to the absorption or transport of various ions. Its regulatory mechanisms under different conditions vary, by activation or inactivation, in a Ca^2+^-dependent or -independent manner, interacting with CBLs or not [31]. All these factors contribute to the specification of AtCIPK23′s role. Which nutrient transporters might be the targets of NtCIPK23 in tobacco? Which CBLs are its interacting partners? Are there any diverse functions in tobacco plants? These questions are far from being answered, and are needed in the future.

*AtCIPK23* was found to be highly expressed in cotyledon, leaves, and radicle in *Arabidopsis* seedlings, but not in hypocotyl [15], which is different from *NtCIPK23*. Phenotypic analysis of *atcipk23* also showed that the absence of *AtCIPK23* does not significantly affect the hypocotyl elongation and seed germination of *A*. *thaliana* [15,20]. All these data hint that *AtCIPK23* might be dispensable during hypocotyl elongation or seedling emergence. Although *AtCIPK23* and *NtCIPK23* are homologous genes with similar nucleotide sequences, due to the different expressional level in hypocotyl, the two genes play different roles in hypocotyl elongation. Therefore, during the functional characterization of homologous genes, enough attention should be paid to the specific intracellular environments, including the expression pattern (species, tissue, organ, cell-type, treatment), upstream or downstream pathways, interactive targets, etc. [37]. On the basis of these differences, genes with high homology might have different functions. The knowledge is very useful in the functional study of an individual gene member from its multigene family, especially when there is functional redundancy. Meanwhile, it was also clearly shown that conclusions from model plants, such as *A*. *thaliana*, could not represent all conditions in plants, and different species have their own characteristics.

Different kinds of phytohormone response, anaerobic response, photoreactive, and stress defense-related elements were found in *NtCIPK23* promoter, which strongly suggests that *NtCIPK23* might be regulated by numerous environmental or cellular factors. Consistently with the prediction, GUS staining assay demonstrated that during the short stage of early seedling growth, obvious expressional changes of *NtCIPK23* occurred spatiotemporally. It hinted that *NtCIPK23* is probably regulated by a vastly complicated network, in which the light, phytohormone, and other kinds of factors are involved. The following RT-qPCR detection also confirmed this prediction, which indicated the regulatory role of light and dark in *NtCIPK23* expression (Figure 5c). As other *CIPK23* genes are proved to occupy a crucial position in nutrition, development, and stress tolerance in plants [3,4,22,38], the upstream regulation pathway of *NtCIPK23* might be an interesting point to be focused on.

It is worth mentioning that hypocotyl elongation is an important process for the epigaeous seedlings. It ensures that the cotyledons are unearthed and reach for light in time [39,40]. All factors involved in this fundamental growth period can directly affect seedling emergence and uniformity. Currently, the latter is given more attention in intensive planting and standardized management [41]. Contributions of *NtCIPK23* to hypocotyl elongation in this study suggested that the gene is of potential agronomic significance in the improvement of seedling emergence and uniformity, and it is quite necessary to deepen the knowledge of *NtCIPK23* in seed germination and early seedling growth.

## 4. Materials and Methods

### 4.1. Plant Materials and Growth Conditions

*N*. *tabacum* L. cv. Zhongyan100 (we refer to it simply as ZY100) and other ZY100 materials with different *NtCIPK23* expression levels were used in this study. During germination and GUS histochemical assay, tobacco seeds were sown on two pieces of filter paper saturated with water, in a culture dish, with vermiculite underlying the filter paper. For the measurement of hypocotyl length and the cotyledon size of tobacco plants, seeds were sown on perforated 96-well PCR plates, which were filled with vermiculite, and saturated with water. Seeds in different treatments were cultivated under constant light at 25 °C ± 1 °C, 60 ± 5% relative humidity. For the dark treatment, the seeds were sown on perforated 96-well PCR plates with vermiculite, saturated with water, and put into boxes wrapped by aluminum foil.

### 4.2. Gene Cloning and Plasmid Construction

Based on the BLAST analysis, one sequence of *AtCIPK23′*s homolog (GenBank No. XM_016594430.1) in *N*. *tabacum* was obtained from NCBI website (https://blast.ncbi.nlm.nih.gov/Blast.cgi), using *AtCIPK23* sequence (At1G30270) as the template. *NtCIPK23* sequence was mapped on Ntab-TN90_scaffold36089 in tobacco genome database (https://www.ncbi.nlm.nih.gov/nuccore). The segments of *NtCIPK23* CDS and its promoter were then cloned from ZY100, based on the design of corresponding primer pairs NtCIPK23-1F/NtCIPK23-1R and NtCIPK23pro-1F/NtCIPK23pro-1R. The CDS segment was used for generation of overexpression lines. PCR products of NtCIPK23 and its promoter were ligated to pMD19-T to obtain pMD19-T-NtCIPK23 and pMD19-T-ProNtCIPK23, respectively.

To construct the expression vector for subcellular localization, *NtCIPK23* segment was amplified from plasmid pMD19-T-NtCIPK23, using the primer pair NtCIPK23-3F-*Nco*I/NtCIPK23-7R-*Sal*I. PCR products were digested with *Nco*I and *Sal*I, and ligated into the *Nco*I/*Sal*I-digested pCambia1300. The plasmid was named as pCambia1300-NtCIPK23-GFP. To generate the overexpressing vector of *NtCIPK23*, pMD19-T-NtCIPK23 (reverse insertion) plasmid was digested by *Sma*I/*Sal*I, and the released segment was ligated into *Sma*I/*Sal*I-digested pCHF3. For the construction of the pBI101-*ProNtCIPK23*::*GUS* vector, the primer pair NtCIPK23pro-2F-*Hin*dIII/NtCIPK23pro-2R-*Bam*HI was used. The PCR product was digested with *Hin*dIII and *Bam*HI and cloned into *Hin*dIII/*Bam*HI-digested pBI101 vector.

The potential guide RNA (gRNA) sequence was initially obtained by CRISPR MultiTargeter (http://www.multicrispr.net/index.html), based on the sequence of *NtCIPK23* CDS. The main principles behind the screening of potential gRNA target were that (1) the binding position of gDNA should be close to the transcription initiation site; (2) the binding position of gRNA should be within the coding frame; and that (3) the gRNA is specific to distinguish NtCIPK23 and its homologous genes in ZY100. Based on the analysis of CRISPR MultiTargeter and the outlined requirements above, a potential primer target (ATGATGTAGGGAGGACCCTTGGG) was obtained. Before the synthesis of gRNA primer, (1) NGG was deleted; (2) one G was added, if the 5′ end was not G; (3) the reverse complemental primer was acquired; and (4) GATT at 5′ end of forward primer and AAAC at 5′ end of reverse primer were also added, respectively. The primer pair NtCIPK23CR-1Target-1F/NtCIPK23CR-1Target-1R of gRNA was obtained. The gRNA expression cassette was then inserted into *Bsa*I-HF (NEB company)-digested pORE-Cas9 binary vector to generate the NtCIPK23-CRISPER/Cas9 vector [42].

The primers used in the experiments are shown in Table S2. All clones derived from the PCR products were verified by sequencing, and the recombinant plasmids were confirmed by restriction analyses.

### 4.3. RNA Extraction, RT-PCR, and Real-Time Quantitative PCR (RT-qPCR) Analyses

To test the expression level of exogenous *NtCIPK23*, total RNA was extracted from the leaves of transgenic plants, using a phenol-based method [31]. cDNA was synthesized from 1 μg total RNA for RT-PCR, using the PrimeScriptTM RT kit (TaKaRa Biotechnology Co., Ltd., Dalian, China). *NtL25* is a ribosomal protein gene (Accession No. L18908), widely used as a common internal control in *N*. *tobacum* [43,44,45]. The primer pairs NtCIPK23-qF/pCHF3-Allcheck-2 and NtL25-F/NtL25-R were used to detect the expression levels of exogenous *NtCIPK23* and relative quantification in RT-PCR [43]. The primer pair NtCIPK23-qF/pCHF3-Allcheck-2 was used to detect the expression levels of exogenous *NtCIPK23* in RT-PCR. The pCHF3-Allcheck-2 is a specific reverse primer antisense to the adjacent sequence, exactly after the multiple cloning sites of transgenic vector pCHF3 (Figure S1). In RT-PCR, only the transcripts of exogenous *NtCIPK23*, but not those of endogenous *NtCIPK23*, were amplified as the templates. The amplification reactions were performed in a total volume of 20 μL, which contained 7.2 μL ddH_2_O, 0.8 μL forward and reverse primers (10 μM), and 2 μL cDNA (diluted 10 times after synthesis), 10 μL 2 × rTaq Mix (TaKaRa Biotechnology Co., Ltd., Dalian, China). PCR was conducted as follows: 95 °C for 3 min, followed by 30 cycles of 95 °C for 30 s and 55 °C for 30 s and 72 °C for 1 min, then 72 °C for 10 min.

To investigate the expressional changes of *NtCIPK23* in the hypocotyl, RT-qPCR was conducted. Total RNA was extracted from the hypocotyl of ZY100 plants treated in the dark or under light (at 6 DAS). The cDNA synthesis method was the same as the above process. The SYBR Premix *Ex* TaqTM (TaKaRa Biotechnology Co., Ltd., Dalian, China) kit was used for quantitative analysis. Specific primer pairs *NtCIPK23*-qF/NtCIPK23-qR and NtL25-F/NtL25-R were used for RT-qPCR and relative quantification, respectively. The mean values of at least three biological replicates were normalized using the *NtL25* gene as the internal controls [45] The amplification reactions were performed in a total volume of 20 μL, which contained 10 μL 2 × SYBR Premix *Ex* TaqTM, 7.2 μL ddH_2_O, 0.8 μL forward and reverse primers (10 μM), and 2 μL cDNA (diluted 10 times after synthesis). PCR was conducted as follows: 95 °C for 1 min, followed by 40 cycles of 95 °C for 10 s and 60 °C for 34 s. Relative quantitative analysis was performed using the standard curve method, and the instrument used was Roche LightCycler 96 Instrument (Roche Molecular Systems, Inc., Basel, Switzerland). Three biological replicates were included for data quantification. The primers used in the experiments are shown in Table S2.

### 4.4. Generation of Transgenic Materials

To generate the *NtCIPK23*-overexpressing lines and *ProNtCIPK23*::*GUS* transgenic plants, pCHF3-NtCIPK23 vector and pBI101-*ProNtCIPK23*::*GUS* vector were transformed into *Agrobacterium* *tumefaciens* EHA105, respectively, and then introduced into *N*. *tabacum* L. cv. Zhongyan100 via the *Agrobacterium*-mediated method [46]. Thirty-four *NtCIPK23-*overexpressing plants and 16 *ProNtCIPK23*::*GUS* transgenic plants were screened out by genomic PCR and RT-PCR/GUS staining. The seeds (T1 generation) of transgenic lines were screened on 1/2 MS medium containing 50 μg/mL kanamycin, and were selectively propagated for T2 generations to obtain the homozygous lines. Seven independent and homozygous T2 overexpressing lines with single copy insertion were finally selected, and 6 lines exhibited similar phenotypes in germination and early seedling growth. Two lines (T2-OE-15-11 and T2-OE-25-4, referred to as OE15 and OE25, respectively) were selected for phenotype analysis. As to the *ProNtCIPK23::GUS* materials, 3 independent and homozygous T2 lines with single copy insertion exhibiting similar expression pattern, were finally obtained. T2-55-13 was selected for expression analysis of *NtCIPK23*.

To obtain loss-of-function materials of *NtCIPK23*, CRISPR/Cas9 system was used for targeted mutagenesis of *NtCIPK23* in ZY100 [42]. The workflow is shown in Figure S1. To generate independent C0 plants, all transgenic seedlings were separated from different tobacco calluses (one seedlings-one callus) and transferred to the rooting medium; 52 C0 plants were obtained. Among these plants, 17 C0 plants were confirmed to be edited via direct sequencing of PCR products, using the specific primer pair NtCIPK23-1-UTR2F/NtCIPK23-1-145R, which could distinguish *NtCIPK23* from other tobacco homologs. Same PCR products were then cloned into pMD19-T vector, and the gene editing events were confirmed by the monoclonal sequencing (clone number > 80). The C0 plants, in which all 80 clones showed the same editing site, were considered to be *NtCIPK23*-edited homozygous lines. There were 6 homozygous and 12 heterozygous plants, respectively. All 6 plants exhibited the same C deletion at the target site, which resulted in a frameshift at the 5′-terminal region of *NtCIPK23* transcripts and finally led to translation termination (Figure S2). The seeds of C0 homozygous seedlings (C1 generation) were obtained individually by self-pollination, and their editing condition was confirmed again by another cycle of sequencing (clone number > 80). The 6 C1 lines showed similar developmental phenotypes, and a typical homozygous line (C1-33#) was designated as the *ntcipk23* mutant and used in the experiments. The primers used in the experiments are shown in Table S2.

### 4.5. GUS Histochemical Assay

Germination of *ProNtCIPK23*::*GUS* seeds occurred within 3 days after sowing (DAS) (denoted as radicle emergence through the seed coat). Seedlings at different growth stages, including the micropylar endosperm rupture, radicle emergence and elongation, hypocotyl elongation, cotyledon emergence and expansion, cotyledon maturation, and emergence and expansion of the first two leaves, were selected for GUS histochemical staining. The samples were completely immersed in GUS staining solution (Lot.1127A19, Beijing Leagene Biotechnology Co., Ltd., Beijing, China) and incubated at 37 °C for 24 h. Afterwards, the chlorophyll of the samples was completely removed with ethanol for the microscope observation. 

### 4.6. Subcellular Localization Assay

The pCambia1300-NtCIPK23-GFP plasmid, PM (Plasma membrane) marker pm-rk *CD3-1007* and pGDp19 were transformed into *A. tumefaciens* EHA105, and were then infiltrated into leaves of *N. benthamiana*, as described previously [29]. Pictures were captured with confocal microscope (Leica TCP SP8, Leica Microsystems, Germany), 48 h after infiltration. The GFP was excited at 488 nm and its emission was captured at 550–590 nm [47]. The mCherry was detected at 543 nm and its emission was captured at 570–600 nm.

### 4.7. Measurement and Statistical Analysis

Radicle protrusion was used as an indicator for seed germination. Green cotyledon percentage was determined to indicate the tobacco post-germination seedling growth. Generally, the radicle breaks through seed coat within 3 DAS. When the radicle began to protrude from the testa, the germination percentage was measured (during 2.5~3.5 DAS). The green cotyledon percentage was calculated when the cotyledon began to turn green (during 3~5 DAS). To measure the cotyledon size of seedlings, mature cotyledons of the seedlings at 8 DAS were sampled and placed on 1/2 MS medium, and the images were taken by an automatic colony counter (Shineso 2.0, Hangzhou Shineso Biotechnology Co., Ltd., Hangzhou, China). To measure the hypocotyl length, the seedlings at 8 DAS were taken out of the 96-well PCR plates and washed gently by water, and pictures of the images were taken. The seedlings required for the measurement of hypocotyl length in the dark (wrapped by aluminum foil) were sampled at 6 DAS. Each experiment was independently performed using three biological repeats with three technical replicates. The number of seedlings for the measurements of green cotyledon percentage, cotyledon size, and hypocotyl length were about 100 seedlings, 24 cotyledons (from 12 seedlings), and 20 hypocotyls for each plant materials in one biological repeat. All seedlings were randomly selected.

Cotyledon area and hypocotyl length were measured by the image processing software ImageJ (https://imagej.nih.gov/ij/). Data obtained by ImageJ were analyzed by one-way ANOVA using the statistical software SPSS 16.0 (https://spss.en.softonic.com/), and were demonstrated by OriginPro 9.0 (https://www.originlab.com/).

## Data Availability

The data presented in this study are available on request from the corresponding author.

## Supplementary Materials

The following are available online at https://www.mdpi.com/2223-7747/10/2/323/s1. Figure S1: The acquisition workflow of the *ntcipk23* mutant; Figure S2: Translation overview of *NtCIPK23* CDS from ZY100 and *ntcipk23*; Figure S3: The GUS staining analysis of *ProNtCIPK23*::*GUS* transgenic tobacco plants during the hypocotyl elongation stage under light and in the dark; Figure S4: The multiple cloning sites of the over-expressing vector pCHF3 and the position of the specific primer pCHF3-Allcheck-2; Table S1: The list of *cis*-acting elements predicted in *NtCIPK23* promoter; and Table S2: Primers used in the experiments.
