# Supplementary material for: The CBL-Interacting Protein Kinase NtCIPK23 Positively Regulates Seed Germination and Early Seedling Development in Tobacco (Nicotiana tabacum L.)
Source: Plants (Basel). 2021 Feb 8;10(2):323. doi: 10.3390/plants10020323 (PMC7915007; doi:10.3390/plants10020323)
Supplement: Supplementary file 1 [file plants-10-00323-s001.pdf]

Supplementary Material

The CBL-interacting protein kinase NtCIPK23 positively regulates seed germination and early seedling development in tobacco (*Nicotiana tabacum* L.)

Sujuan Shi [1,2,3,†], Lulu An [1,2,†], Jingjing Mao [1,2], Oluwaseun Olayemi Aluko [1,2], Zia Ullah [1,2], Fangzheng Xu [1,2], Guanshan Liu [1], Haobao Liu [1,*] and Qian Wang [1,*]

[1] Tobacco Research Institute, Chinese Academy of Agricultural Sciences, Qingdao 266101, China; shisujuan2014@163.com (S.S.); lulu_an9@163.com (L.A.); maojingjing40@163.com (J.M.); aluko.oluseun@gmail.com (O.O.A.); zianust512@gmail.com (Z.U.); xufangzheng@caas.cn (F. X.) liuguanshan@caas.cn (G.L.)

[2] Graduate School of Chinese Academy of Agricultural Sciences (CAAS), Beijing 100081, China

[3] Technology Center, Shanghai Tobacco Co., Ltd., Beijing 101121, China

* Correspondence: liuhaobao@caas.cn (H.L.); wangqian01@caas.cn (Q.W.); Tel.: +86-0532-8870-1031 (H.L. & Q.W.)

[†] These authors contributed equally to this work.

List of Supplementary Material

Figure S1. The acquisition workflow of the *ntcipk23* mutant.

Figure S2. Translation overview of *NtCIPK23* CDS from ZY100 and *ntcipk23*.

Figure S3. The GUS staining analysis of *NtCIPK23* in the hypocotyl of tobacco seedlings.

Figure S4. The multiple cloning sites of the over-expression vector pCHF3 and the position of the specific primer pCHF3-Allcheck-2.

Table S1. Primers used in the experiments.

Table S2. The list of *cis*-acting elements predicted in *NtCIPK23* promoter.

Figure S1

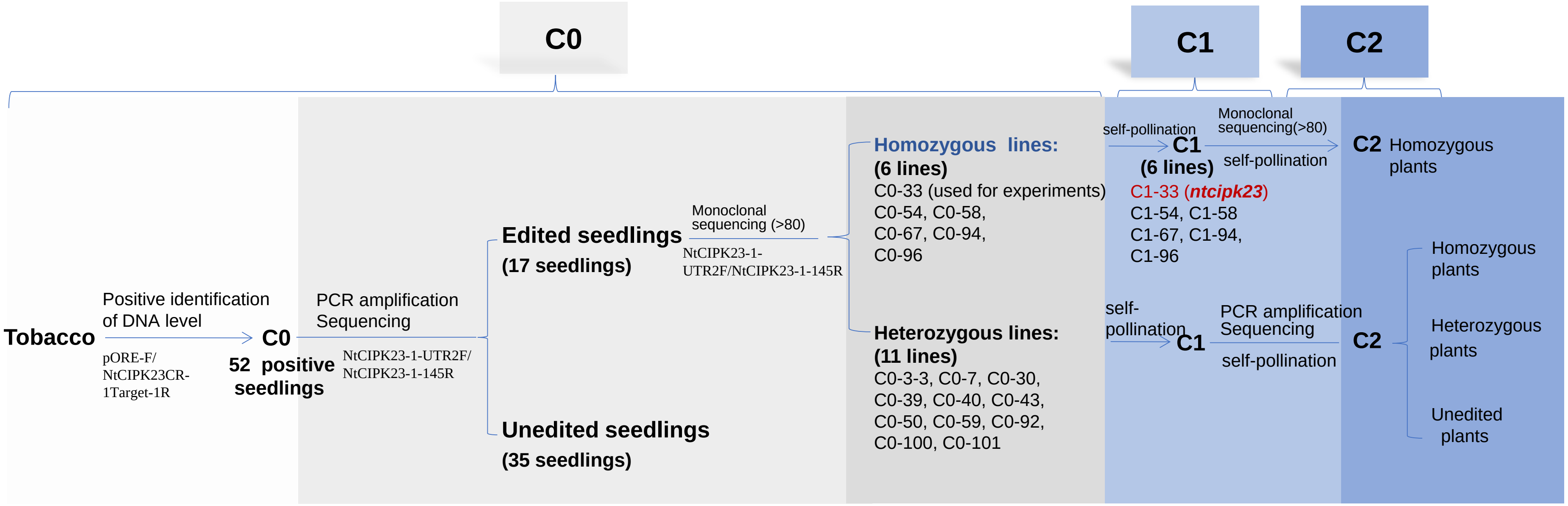

Figure S1. The acquisition workflow of the *ntcipk23* mutant. 52 C0 plants were obtained and among these plants, 17 C0 plants were verified to be edited *via* PCR product sequencing, using primer pair NtCIPK23-1-UTR2F/NtCIPK23-1-145R. Same PCR products were then cloned into pMD19-T vector, and the gene editing events were confirmed by the sequencing of multiple clones (clone number > 80). The C0 plants, in which all the 80 clones showed the same editing site, were considered to be the homozygous lines. Finally, there were 6 homozygous and 12 heterozygous plants, respectively. All the 6 plants exhibited the same C deletion at the target site. The seeds of C0 homozygous seedlings (C1 generation) were obtained individually by strict selfing, and their editing condition was confirmed again by another cycle of sequencing (clone number > 80). The six C1 lines showed similar developmental phenotypes, and a typical homozygous line (C1-33#) was designated as the *ntcipk23* mutant, and its seedlings were used in the experiments.

Figure S2

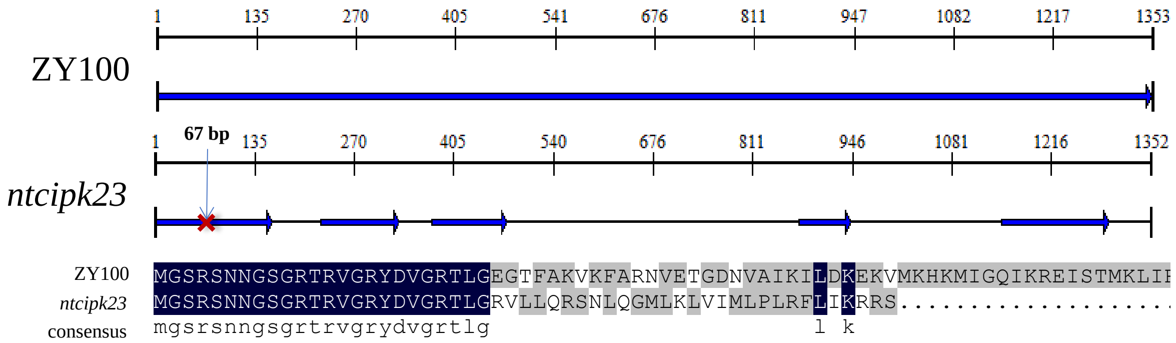

Figure S2. Translation overview of *NtCIPK23* CDS from ZY100 and *ntcipk23*. The C deletion at position 67 in *NtCIPK23* CDS of the *ntcipk23* plants results in a frameshift at the 5′-terminal region of *NtCIPK23* transcripts and finally leads to translation termination.

Figure S3

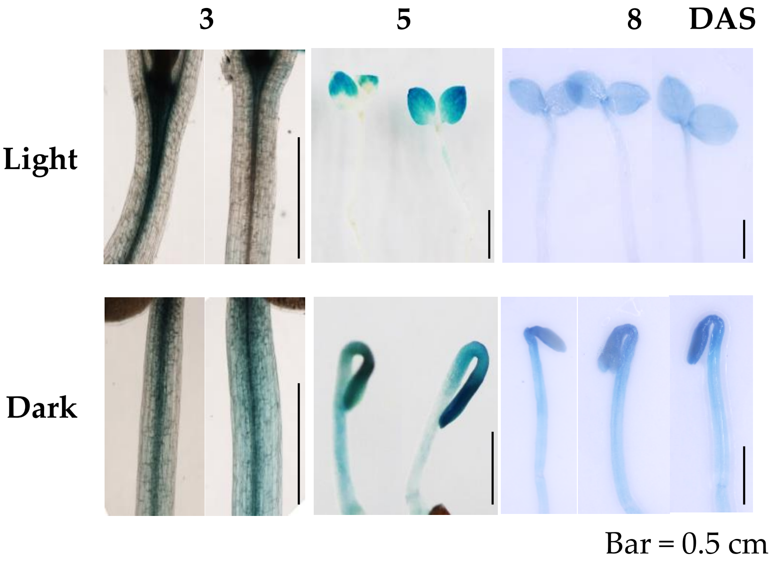

Figure S3. The GUS staining result at 3,5 and 8 DAS of *ProNtCIPK23*::*GUS* transgenic plants under light and dark conditions. Scale bar is 0.5 cm.

Figure S4

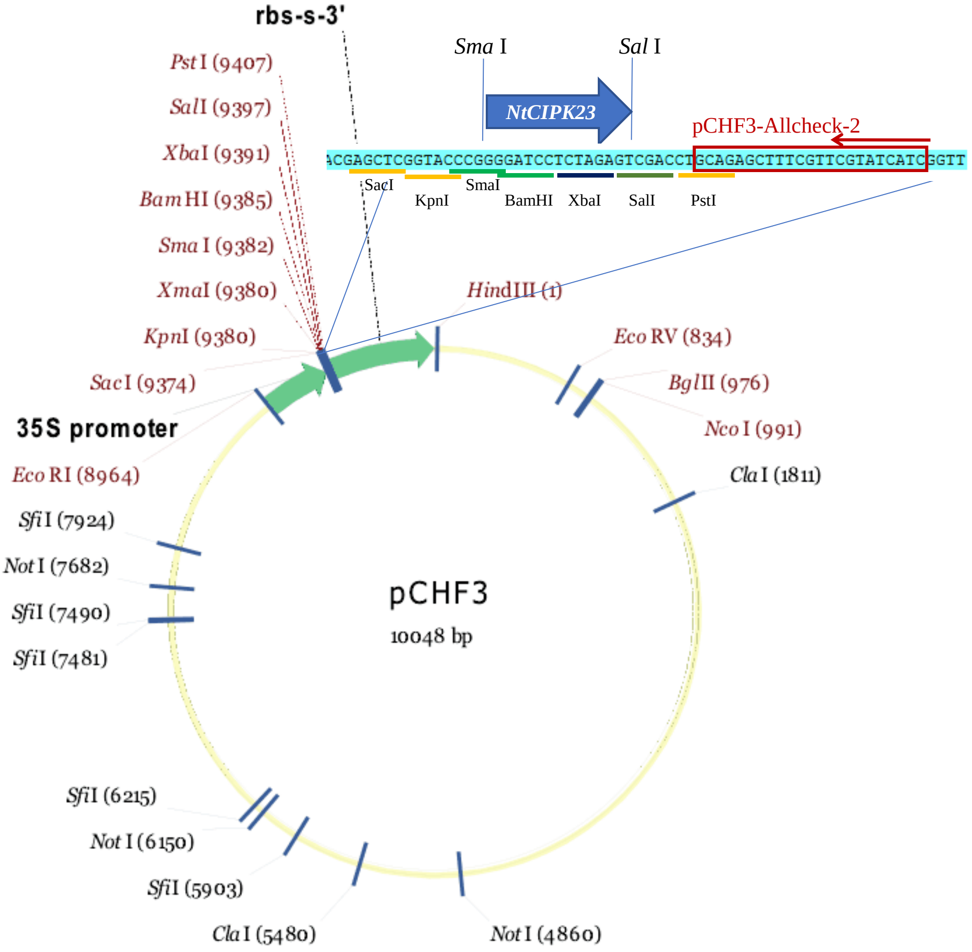

Figure S4. The multiple cloning sites of the over-expression vector pCHF3 and the position of the specific primer pCHF3-Allcheck-2. The primer pCHF3-Allcheck-2 for distinguishing exogenous and endogenous *NtCIPK23* transcripts in RT-PCR is shown in red box. The pCHF3-Allcheck-2 was designed based on the 23 bp-sequence right after the multiple cloning sites of pCHF3 vector.

Table S1. The list of *cis*-acting elements predicted in *NtCIPK23* promoter

Element Type	Site Name	Numbe	Position	Core Sequence	Function
**Core element**	TATA-box	37	+292/-1900/-	TAAAGATT	core promoter element around -30 of transcription start
**Enhancer**	CAAT-box	39	-476/-588/+623/…	CAAT	common *cis*-acting element in promoter and enhancer
**Hormone response element**	ABRE	3	+365/+1155/+366	CACGTG	*cis*-acting element involved in the abscisic acid
	CGTCA-motif	1	+801	CGTCA	*cis*-acting regulatory element involved in the MeJA-
	TGACG-motif	1	-801	TGACG	*cis*-acting regulatory element involved in the MeJA-
	ERE	1	-834	ATTTTAAA	*cis*-acting element involved in ethylene-responsivness
	P-box	3	+1529/+1701/-1672	CCTTTTG	gibberellin-responsive element
	GARE-motif	1	+42	TCTGTTG	gibberellin-responsive element
	TATC-box	1	-28	TATCCCA	*cis*-acting element involved in gibberellin-responsiveness
	TCA-element	2	+1456/+1501	CCATCTTTTT	*cis*-acting element involved in salicylic acid responsiveness
	TGA-element	1	-758	AACGAC	auxin-responsive element
**Light response element**	Box 4	2	+989/-1179	ATTAAT	part of a conserved DNA module involved in light
	G-Box	2	+365/+1154	ATTTTAAA	*cis*-acting regulatory element involved in light
	GATA-motif	1	+856	GATAGGG	part of a light responsive element
	Sp1	1	-1399	GGGCGG	light responsive element
	TCT-motif	1	-1979	TCTTAC	part of a light responsive element
**Defense and stress response element**	ARE	2	+619/-1684	AAACCA	*cis*-acting regulatory element essential for the anaerobic
	W box	3	-392/+907/+609	TTGACC	disease resistance.
	TC-rich repeats	2	-90/+1285	ATTCTCTAA	*cis*-acting element involved in defense and stress
	as-1	1	-801	TGACG	stress responsiveness

Table S2. Primers used in the experiments.

Primer	Sequence (5′ to 3′)	Description
NtCIPK23-1F	GGCATGGGTTCAAGATCAAATAATGG	Used for the cloning of *NtCIPK23* CDS sequence.
NtCIPK23-1R	CGCTCGCCTAACCATCTTTTAC	
NtCIPK23pro-1F	AAGCTTGAGGCTTCTGCTGGTTGGAG	Used for the cloning of *NtCIPK23* promoter sequence.
NtCIPK23pro-1R	GGATCCCTACCTCCAAACTTTCTATTTCTT	
NtCIPK23-3F-*Nco*I	CATGCCATGGGCATGGGTTCAAGATCAAA	Used for the construction of pCambia1300-NtCIPK23-GFP vector.
NtCIPK23-7R-*Sal*I	ACGCGTCGACGCATAACCATCTTTTAC	
NtCIPK23CR-1Target-1F	**GATT**GTGATGTAGGGAGGACCCTTG	Used for the construction of NtCIPK23-CRISPER/Cas9 vector.
NtCIPK23CR-1Target-1R	**AAAC**CAAGGGTCCTCCCTACATCA	
NtCIPK23pro-2F-*Hind*III	CCCAAGCTTGAGGCTTCTGCTGGTTGGAG	Used for the construction of pBI101- ProNtCIPK23::GUS vector.
NtCIPK23pro-2R- *Bam*HI	CGGGATCCCTACCTCCAAACTTTCTATTTC	
NtCIPK23-1F	GGCATGGGTTCAAGATCAAATAATGG	Used for the positive screening of *NtCIPK23* overexpressing materials
pCHF3-R	ATTCTGGTGTGTGCGCAATG	
pBI101-F	CCGATTCATTAATGCAGCTG	Used for the positive screening of *NtCIPK23* promoter transgenic material
NtCIPK23pro-2R	GGATCCCTACCTCCAAACTTTCTATTTCTT	
pORE-F	TTAGGTTTACCCGCCAATA	Used for the positive detection ofNtCIPK23-CRISPER/Cas9 transgenic
NtCIPK23-1-UTR2F	ACAAGAGGATGGGATTTGT	Used to detect the edition sites of *NtCIPK23*
NtCIPK23-1-145R	ATCACCAGTTTCAACATTCC	
NtCIPK23-qF	CCACTGACTATGAATGCTTT	Used for *NtCIPK23* amplification in qPCR
NtCIPK23-qR	GCGCCCACTCTTCTCCCCA	
NtL25-qF	CAAAAGTTACATTCCACCG	Used for *NtL25* amplification in RT-PCR and qPCR
NtL25-qR	TTTCTTCGTCCCATCAGGC	

NtCIPK23-qF	CCACTGACTATGAATGCTTT	Used to detect the expression level of exogenous *NtCIPK23* in RT-PCR
pCHF3-Allcheck-2	GATGATACGAACGAAAGCTCTGC	

Note: The F or R designation in the primer names denotes whether the primer is a forward (5′) or reverse primer (3′), respectively. The nucleotides underlined and those in bold denote the restricted enzyme sites and primer adaptor, respectively.
